# The influence of psychological and cognitive states on error-related negativity evoked during post-stroke rehabilitation movements

**DOI:** 10.1186/s12938-021-00850-2

**Published:** 2021-02-02

**Authors:** Akshay Kumar, Qiang Fang, Elena Pirogova

**Affiliations:** 1 School of Engineering, Royal Melbourne Institute of Technology University, Melbourne, Australia; 2 Department of Biomedical Engineering, College of Engineering, Shantou University, Guangdong, China

**Keywords:** Assist-as-needed, Brain–computer interface (BCI), Cognitive, Error-related negativity (ERN), Error-related potential (ErrP), Psychological, Stroke rehabilitation, Training-ERN, Training-ErrP

## Abstract

**Background:**

Recently, error-related negativity (ERN) signals are proposed to develop an assist-as-needed robotic stroke rehabilitation program. Stroke patients’ state-of-mind, such as motivation to participate and active involvement in the rehabilitation program, affects their rate of recovery from motor disability. If the characteristics of the robotic stroke rehabilitation program can be altered based on the state-of-mind of the patients, such that the patients remain engaged in the program, the rate of recovery from their motor disability can be improved. However, before that, it is imperative to understand how the states-of-mind of a participant affect their ERN signal.

**Methods:**

This study aimed to determine the association between the ERN signal and the psychological and cognitive states of the participants. Experiments were conducted on stroke patients, which involved performing a physical rehabilitation exercise and a questionnaire to measure participants' subjective experience on four factors: motivation in participating in the experiment, perceived effort, perceived pressure, awareness of uncompleted exercise trials while performing the rehabilitation exercise. Statistical correlation analysis, EEG time-series and topographical analysis were used to assess the association between the ERN signals and the psychological and cognitive states of the participants.

**Results:**

A strong correlation between the amplitude of the ERN signal and the psychological and cognitive states of the participants was observed, which indicate the possibility of estimating the said states using the amplitudes of the novel ERN signal.

**Conclusions:**

The findings pave the way for the development of an ERN based dynamically adaptive assist-as-needed robotic stroke rehabilitation program of which characteristics can be altered to keep the participants’ motivation, effort, engagement in the rehabilitation program high. In future, the single-trial prediction ability of the novel ERN signals to predict the state-of-mind of stroke patients will be evaluated.

## Background

In recent years, stroke has become the leading cause of disability in the world [1]. With the rapid growth in the number of stroke patients worldwide, there is an urgent need for efficient rehabilitation approaches [1]. Recently, assist-as-needed (AAN) robotic stroke rehabilitation programs have gained popularity because of their potential ability to conduct rehabilitation programs autonomously and provide assistance to stroke patients in performing the rehabilitation exercises only when required [2, 3]. However, a recent study by Rodgers et al. [4] reported that AAN robot therapies delivered using the MIT-Manus robotic gym did not show any significant improvement in upper-limb functional abilities in comparison to standard stroke rehabilitation care, the latter which is 45 min of appropriate rehabilitation therapy for a minimum of 5 days per week.

Error-related potential (ErrP) is an event-related potential signal that is elicited in the human brain following the perception of an error [5, 6]. In a recently reported study, it was shown that ErrP signals could be observed in a *new task situation*, i.e., when stroke patients were performing physical rehabilitation exercises, called training-ErrP onwards [7]. The training-ErrP signal was proposed for the development of a *human-in-the-loop* AAN robotic stroke rehabilitation system, which can modulate the robotic assistance level in real time using the brain’s intrinsic feedback mechanism [7]. The amount of time spent in actively performing the rehabilitation exercises is positively linked with the rate and amount of recovery from stroke disabilities [8]. As adaptive rehabilitation programs have shown to promote prolonged training sessions and engagement in the rehabilitation programs [9, 10], a training-ErrP-led *human-in-the-loop* AAN robotic stroke rehabilitation program can provide a higher rate of recovery to stroke patients from their motor impairments in comparison to the existing state-of-the-art AAN robotic stroke rehabilitation programs and standard stroke rehabilitation care [4, 7, 11].

The error-related negativity (ERN) signal of a typical ErrP signal, called typical-ERN hereafter, has two major components: negative-going deflection (Ne) at approximately 50–200 ms following the perception of an error, followed by a positive activity (Pe) at ~200–500 ms [11]. To date, several studies have investigated the effects of various physical and psychological states of human participants on the amplitude of their typical-ERN signals [12–15]. Maruo et al. [16] showed how the motivational significance of the committed error affects the amplitude of the ERN signal. Specifically, larger ERN amplitudes were observed when errors were important for the participants. Similarly, Hajcak et al. [17] showed larger ERN amplitudes in trials carrying larger rewards. Peters et al. [18] showed that higher anxiety is linked with larger ERN amplitudes, and that depressive symptoms attenuate ERN amplitudes. Moore et al. [19] showed that mental fatigue built over time, and decreased attention, result in reduced ERN amplitudes. Apart from these, other factors such as age [14], personality traits [20], ethnicity [14] and even gender [14] affect the typical-ERN signal. Notably, due to the association between the typical-ERN signals and the psychological states mentioned earlier, the typical-ERN signals can serve as a predictor for the psychological states [13].

A number of studies have reported that psychological states, such as motivation and pressure to participate in the rehabilitation program, affect a patient’s engagement in the rehabilitation program [15, 19, 21]. Previous studies linked the patient’s engagement in the rehabilitation program with the rate of recovery from motor impairments [22]. Moreover, a number of studies have also demonstrated the association between the effort in performing the rehabilitation exercises and the rate of recovery from motor impairment [23, 24]. Hence, if parameters of the physical rehabilitation program, such as the exercise type, its difficulty level, and assistance level given, are modified not only based on the presence and absence of ERN signal associated with a training-ErrP signal (named training-ERN onwards), as proposed in [7, 11], but also on patients’ engagement and effort level, a dynamically adaptive stroke rehabilitation system can be developed which will keep stroke patients engaged in the rehabilitation program. Furthermore, any change in the training-ERN signal due to the change in the state-of-mind of a participant can increase the inter-participant as well as inter-trial variability, which can reduce the detection rate of the training-ERN signal. This, in turn, will ultimately reduce the efficiency of the training-ErrP-led AAN robotic stroke rehabilitation system. Nevertheless, with prior knowledge of the participant’s state-of-mind and understanding of its effect on the training-ERN signal, the parameters of the training-ERN detector can be adjusted to accommodate state-of-mind changes of the participant. Nevertheless, before developing such a system, it is imperative to understand how the states-of-mind of participants can affect their training-ERN signal. This problem has not been studied yet.

To address this issue, in this study, the effect of stroke patients’ psychological and cognitive states on the training-ERN signal are reported. In essence, this study investigated:

i. the effects of psychological and cognitive states of stroke patients, specifically, motivation to participate in the rehabilitation program, perceived effort, perceived pressure, and awareness of uncompleted exercise trials (hereafter, the four states are collectively called MEPA states), on the amplitude of training-ERN signal;
ii. the possibility of predicting the level of the MEPA states of stroke patients using the amplitudes of their training-ERN signals.

Here, the awareness of uncompleted exercise trials states a cognitive state of the patients. We hypothesized that the amplitude of the patients’ training-ERN signal would correlate with the level of the four MEPA states. The findings from this study will contribute towards the development of a dynamically adaptive AAN robotic stroke rehabilitation system, for which characteristics can be altered to achieve maximum patient motivation, effort and engagement in the rehabilitation program, which can enhance the neuroplasticity of the brain and improve the rate of recovery of stroke patients.

## Methods

### Experiment protocol and EEG data acquisition

**Fig. 1:**
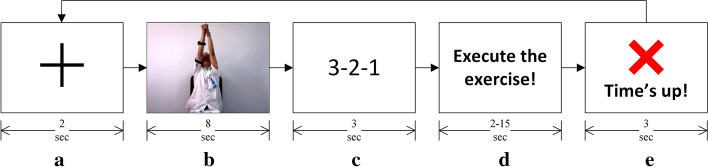
Experimental timeline and visual stimuli of an exercise trial. Instructions were delivered in the following order. **a** A fixation cross marking the start of a trial, **b** exercise video, **c** a 3–2–1 timer, **d** instruction to the participant to start performing the exercise, **e** participants were asked to complete the exercise before this screen appeared

Fifteen stroke patients (5 female, mean age: 57.5 ± 11.3 years) participated in this study. The inclusion criteria for participation were: (1) age over 18; (2) upper-limb motor impairment resulting from an ischemic or hemorrhagic stroke; (3) first ever and single stroke, between 1 week and 6 months of the experiment; (4) moderate-to-severe upper-limb impairment due to stroke (i.e., Brunnstrom stage I–IV); (5) no cognitive impairments; (6) able and willing to give consent and to comply with the requirements of the protocol. Among the 15 stroke patients, two had Brunnstrom stage I, eight had stage II, one had stage III, and four had stage IV movement in their upper-limb. The experiment of this study was approved by the ethics committee of the 2nd Hospital of Jiaxing, China, and the experiment was conducted in accordance with the declaration of Helsinki. In the experiment, patients performed a standard Bobath’s rehabilitation exercise: shoulder flexion–extension while adjoining both hands. The experimental timelines are shown in Fig. 1. A fixation cross (see Fig. 1a) marked the start of a trial. Participants were asked to start performing the rehabilitation exercise depicted in Fig. 1b once the 3–2–1 timer (see Fig. 1c) finished and the instruction shown in Fig. 1d showed up. Participants were asked to complete the exercise before the ‘Time’s up!’ screen (see Fig. 1e) appeared. The trials in which participants completed the exercise before the ‘Time’s up!’ screen appeared were marked as *correct trials*, and the trials in which they were unable to complete the exercise before the ‘Time’s up!’ screen appeared were marked as *error trials*. Each participant participated in at least one session of the experiment containing 24 trials each, out of which about 35% of the trials were *error trials*. The data of Patients 1 and 9 had to be excluded from the analyses because Patient 1 aborted the experiment at the start and Patient 9 did not perform the experiment as per the instructions given. While the participants were performing the rehabilitation exercise (the experiment), 58 channels of monopolar EEG were recorded using an active EEG electrode system and g.HIamp amplifier (g.tec GmbH, Austria) as per the international 10–20 system. For detailed information on the experiment protocol, participants’ clinical information, and data acquisition, see [7].

### EEG data pre-processing

**Fig. 2:**
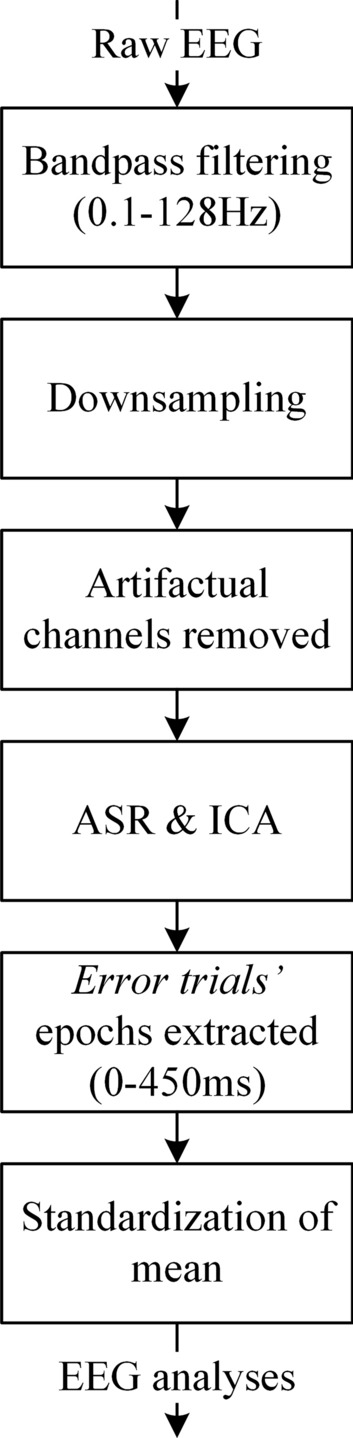
Step-by-step methodology employed to pre-process the raw EEG data before carrying out the analyses

**Fig. 3:**
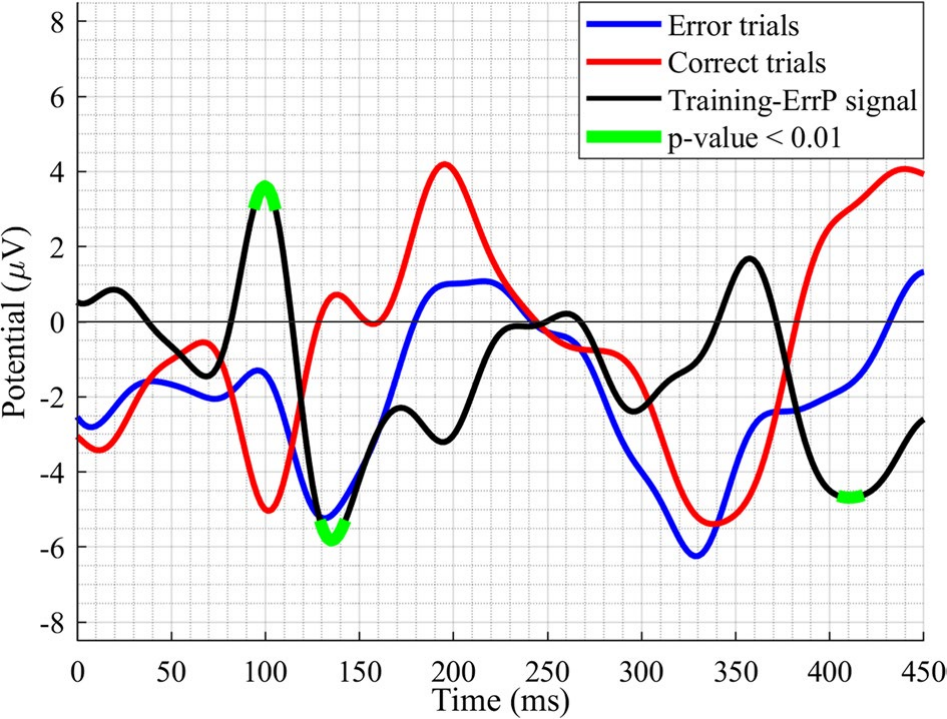
Grand-average waveforms of *error trials* (training-ERN signals, in blue), *correct trials* (in red) and training-ErrP signals (in black) at Cz electrode. The waveforms were smoothed out with a 25-Hz low-pass FIR filter, for an illustration purpose. Training-ErrP signal (in black) is the difference of the neural responses of *error trials* and *correct trials*. Green highlights show the time-points where the training-ErrP signal is significantly different against zero (*p* < 0.01, one-sample *t* test)

A series of pre-processing steps were performed offline on the raw EEG data using MATLAB and EEGLAB [26]-based custom scripts to prepare the data for further analysis. The step-by-step methodology employed to pre-process the raw EEG data before carrying out the analyses is depicted in Fig. 2. Data were bandpass filtered in the range 0.1–128 Hz using a Windowed sinc finite impulse response filter (zero phase shift) to remove low-frequency drifts and high-frequency noise, as per the parameters recommended in [27]. Then the data were down-sampled to 512 Hz, and artifactual channels were removed with manual inspection. Artifact Subspace Reconstruction (ASR) [28] and Independent Component Analysis (ICA) [26] were used to remove transients, and stereotypical and non-stereotypical artifacts, primarily ocular, muscular and cardiac artifacts. After that, the continuous data were segmented into epochs ranging from 0 to 450 ms relative to the onset of Fig. 1e. Only *error trials* were kept for further processing, as they represent the ERN signals [11]. Individual epoch means were subtracted from the respective epochs to remove any DC offset left. Further analyses were carried out on the pre-processed data epochs. The grand-average waveforms of *error trials*, *correct trials* and training-ErrP signals at the Cz electrode location are shown in Fig. 3. The training-ErrP signal (in black in Fig. 3) is the difference of the neural responses of *error trials* (in blue in Fig. 3) and *correct trials* (in red in Fig. 3). The training-ErrP waveform has been highlighted in green at time points where the signal is significantly different against zero (*p* < 0.01, one-sample *t*-test), which essentially exhibits the time points where the *error trials’* neural responses are significantly different from the *correct trials’* neural responses.

### Assessment of psychological and cognitive states

Psychological states of the stroke patients were assessed with the Intrinsic Motivation Inventory (IMI) questionnaire developed by Ryan et al*.* [25], which is a multidimensional assessment tool with seven scales to assess participants’ subjective experiences related to a targeted activity in an experiment. The seven scales are the interest/enjoyment, effort/importance, perceived competence, perceived choice, felt pressure/tension, value/usefulness, and personal relatedness scales, thus yielding seven individual subscale scores while performing a given activity. The IMI questionnaire was adapted to the experiment and was employed to measure participants' subjective experience on the three subscales: *interest/enjoyment*, *effort/importance*, *pressure/tension*. The *interest/enjoyment* is considered as the self-reported measures of intrinsic motivation; *pressure/tension* is considered as the self-reported measures of stress level, which is a negative predictor of intrinsic motivation; and *effort/importance* is a separate variable relevant to some motivation questions [25]. The adapted version of the IMI questionnaire contained 17 statements, as listed in Table 1. In addition, three statements, as listed in Table 2, were included to assess the cognitive state of the participants, by assessing participants’ awareness of the trials in which they failed in completing the rehabilitation exercise in the given time (i.e., *error trials*). Hereafter, the cognitive state is called the *awareness of error* state. The statements listed in Tables 1 and 2 were unified randomly and then used.

**Table 1:** All statements of adapted version of intrinsic motivation inventory questionnaire (Ryan et al*.* [25])

Item	Statement	Factor
1	I enjoyed doing this activity very much	I
2	I put my maximum effort to maintain the accuracy and complete the exercise within time	E
3	I did not feel nervous at all while doing this activity	P^a^
4	I think this was a boring activity	I^a^
5	I tried very hard to complete the exercises on time	E
6	I was very relaxed while doing this activity	P^a^
7	I would describe this activity as very interesting	I
8	It was important to me to do well at this task	E
9	This activity was fun to do	I
10	I felt very uneasy while doing this activity	P
11	I did not put much energy into this task	E^a^
12	I thought this activity was quite enjoyable	I
13	This activity did not hold my attention at all	I^a^
14	I was anxious while working on this activity	P
15	I did not try very hard to do well at this activity	E^a^
16	I felt like I was enjoying the activity while I was doing it	I
17	I felt pressured while doing these	P

The Factor column describes to which subscale the respective statement belongs to, I = interest/enjoyment, E = perceived effort/importance, P = perceived pressure/tension

^a^Scores of these statements were subtracted from 8, before being used for taking an average

**Table 2:** Statements used to assess the *awareness of error* state

Item	Statement
1	I am sure that in some trials I did not complete the exercise on time
2	I am fully aware of the trials in which I failed to complete the exercise on time
3	I know in some trials I did not perform the exercises accurately

At the end of the experiment, participants were asked to fill in the questionnaire to state the status of the emotions they felt during the experiment on a seven-point Likert scale (1: not at all true and 7: very true). All questionnaires were filled out by the participants themselves if they were able to hold a pen or otherwise by one of the experimenters. This assessment lasted 10–20 min. For every participant, a score for each of the four-subscales was obtained by averaging of the scores given on statements belonging to a subscale. In this way, the scores (levels) of the MEPA states of the participants during the experiment were estimated. These scores were then used for further analyses.

### Correlation analysis

The training-ERN signal associated with the training-ErrP signal contains three peaks: a positive-going deflection at 100 ms (P100); followed by a negative-going deflection at 135 ms (N135); and lastly, a larger negative peak at 410 ms (N400); see Fig. 3 [7]. Amplitude values of the three peaks were noted for all participants’ *error trials* from the pre-processed EEG data. Subsequently, for all 13 participants, an average value for each of the three peaks was calculated by averaging the peak amplitude values of all the *error trials* belonging to a participant, which were used for further analyses.

Statistical correlation analysis was carried out to assess the effects of the scores of the MEPA states on the amplitude of the training-ERN signal. However, before the statistical analyses, the Shapiro–Wilk test (α = 0.05) was used to verify the normal distribution of the amplitude of the training-ERN signal and the scores of the MEPA states of the 13 participants [29]. The null hypothesis of Shapiro–Wilk assumes a normal distribution, while the alternative hypothesis denies that. Skewness and kurtosis z-value were used as additional measures to confirm the normality of the average data samples [30]. The null hypothesis, i.e., the assumption of normal distribution, was not rejected for all but N135 amplitude values. The p-values were found to be higher than 0.05; and in addition, skewness and kurtosis *z*-values were within the ± 1.96 range, further strengthening the assumption of normal distribution. Therefore, a parametric measure of correlation, the Pearson correlation coefficient, was used to evaluate the correlation between the MEPA states of the participants and the training-ERN signal. An upper and lower limit of 95% confidence interval (CI) was also estimated using bootstrap distribution repeated over 10,000 times. All statistical analyses were conducted using IBM SPSS Statistics 26.

## Results

**Fig. 4:**
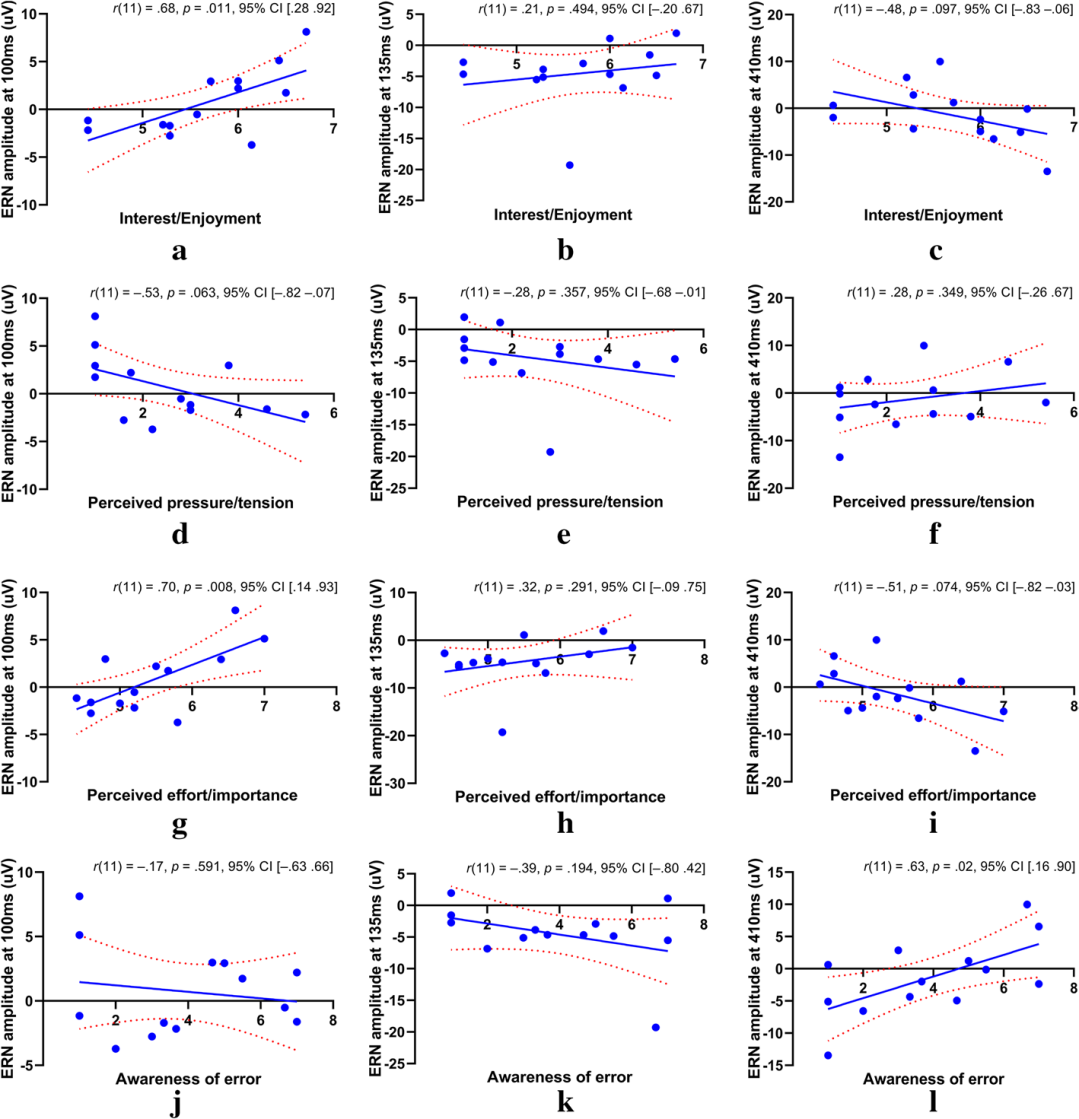
Correlation measurements between the amplitude of peaks of the training-ERN signal and the psychological and cognitive states of the participants. The solid blue lines in the scatter plots show the linear-regression lines, and the dotted red curves show the upper and lower limit of 95% confidence interval (CI) estimated using bootstrap distribution repeated over 10,000 times. The *x*-axis of the figures represents the scores (levels) of the MEPA states of the participants, ranging from 1 to 7

With the help of scatter plots and linear-regression lines, the relationships among the three peaks of the training-ERN signal and the considered psychological and cognitive states (MEPA) are depicted in Fig. 4.

Figure 4a–c shows the scatter plots illustrating the effect of *interest/enjoyment* score on the P100, N135 and N400 training-ERN peaks (respectively). A higher *interest/enjoyment* score has been associated with stronger ERN response, i.e., more positive P100, $r(11)=0.68, p=0.011, 95\mathrm{\% CI }\left[0.28 0.92\right]$ and more negative N400,$r(11)=-.48, p=0.097, 95\% \mathrm{CI} [-0.83 -0.06]$. The $\alpha$ level for statistical significance has been set to 0.017 after correcting for multiple comparisons.

Figure 4d–f shows the scatter plots illustrating the effect of perceived *pressure/tension* score on the P100, N135 and N400 training-ERN peaks (respectively). A higher perceived *pressure/tension* score has been associated with weaker ERN response, i.e., more negative P100, $r(11)=-0.53, p=0.063, 95\mathrm{\% CI\,}\left[-0.82 -0.07\right]$ and more positive N400,$r(11)=0.28, p=0.349, 95\% \mathrm{CI}\,[-0.26 0.67]$.

Figure 4g–i shows the scatter plots illustrating the effect of perceived *effort/importance* score on the P100, N135, and N400 training-ERN peaks (respectively). A higher perceived *effort/importance* score has been associated with stronger ERN response, i.e., more positive P100, $r\left(11\right)=0.70, p=0.008, 95\mathrm{\% CI }\,\left[0.14 0.93\right]$ and more negative N400,$r(11)=-0.51, p=0.074, 95\% \mathrm{CI}\,[-0.82 -0.03]$.

Figure 4j–l shows the scatter plots illustrating the effect of *awareness of error* score on the P100, N135, and N400 training-ERN peaks (respectively). A higher *awareness of error* score has been associated with a mixed ERN response, i.e., stronger ERN or more negative N135, $r(11)=-0.39, p=0.194, 95\mathrm{\% CI }\,\left[-0.80 0.42\right]$ and weaker ERN or more positive N400, $r(11)=0.63, p=0.02, 95\% \mathrm{CI}\,[0.16 0.90]$.

**Fig. 5:**
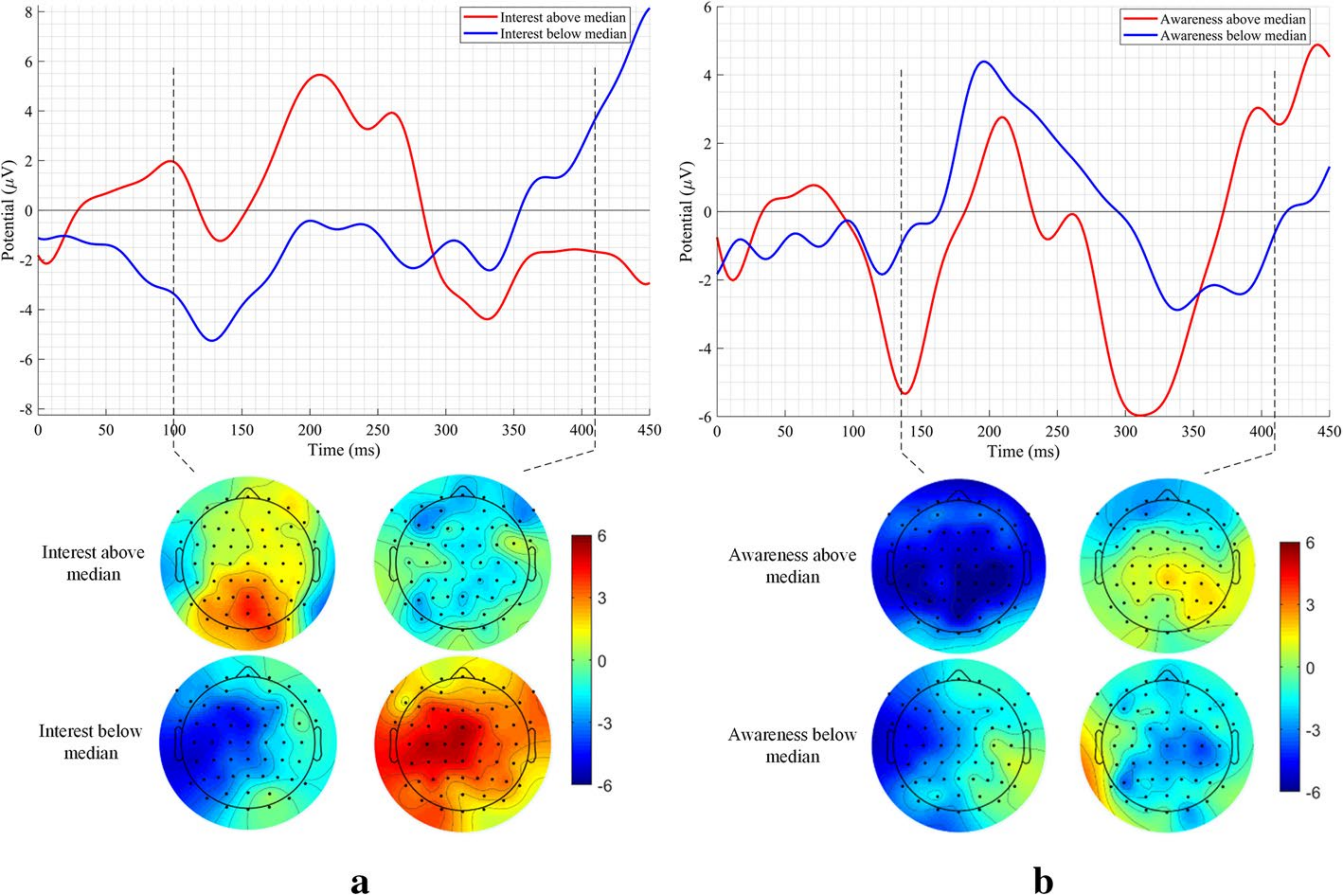
The averaged training-ERN waveforms at the Cz electrode location and the topographical maps of the *above median* and *below median* groups for **a** *interest/enjoyment* state and **b** *awareness of error* state

To carry out further analyses, the median values of the scores of all four MEPA states were noted, and patients were subdivided into two groups: one group of patients having the scores of the MEPA state under consideration, higher than the median value (named *above median* onwards); and the second group having the scores lower than the median value (named *below median* onwards). Both the *above median* and *below median* groups consisted of six patients each. Afterwards, the training-ERN waveforms and topographical maps of the two groups were compared at the three ERN peaks. The averaged training-ERN waveforms at the Cz electrode and the topographical maps of the two groups for *interest/enjoyment* and *awareness of error* state are shown in Fig. 5a, b, respectively. As observed in the results presented so far (Fig. 4), the *interest/enjoyment* state and *awareness of error* state have a weak correlation with N135 and P100 training-ERN peaks, respectively; as a result, the ERN waveforms were only compared at the other two peaks for the two states.

It is to be noted that the ERN waveforms and topographical map comparisons of *above median* and *below median* groups were only carried out for *interest/enjoyment* and *awareness of error* states. In the case of perceived *pressure/tension*, none of the correlations shows significance even before the correction for multiple comparisons. In the case of perceived *effort/importance*, more than one patients were having the states’ scores equal to the median value, which made it difficult to divide the groups in the *above median* and *below median* groups with the minimum number of six trials required to get reliable results [31].

An independent-samples *t*-test was performed to evaluate whether the difference in the *interest/enjoyment* and *awareness of error* scores, by means of *above median* and *below median* group, causes a statistically significant difference in training-ERN peaks’ amplitudes. Before carrying out the independent-samples *t* test, the Shapiro–Wilk test, as well as skewness and kurtosis *z*-value, were used to confirm the normal distribution of the ERN peaks. In line with the trend observed in Fig. 4, the *above median* *interest/enjoyment* score group showed a significantly stronger (at $\alpha =0.05$) training-ERN response $(M=2.74, \mathrm{SD}=3.95)$ in comparison to the *below median* *interest/enjoyment* score group $\left(M=-1.65, \mathrm{SD}=0.78\right), t\left(10\right)=2.68, p=0.023$ for P100 peak (see Fig. 5a; Table 3). Similarly, for N400 peak also, the *above median* *interest/enjoyment* score group showed a significantly stronger training-ERN response $(M=-5.43, \mathrm{SD}=4.55)$ in comparison to the *below median* *interest/enjoyment* score group $\left(M=2.28, \mathrm{SD}=5.35\right), t\left(10\right)=-2.69, p=0.023$ (see Fig. 5a; Table 3). A similar trend is also evident from the central regions of the topographical maps shown in Fig. 5a. Similarly, in line with the mixed-trend observed in Fig. 4, the *above median awareness of error* score group showed a stronger training-ERN response $(M=-6.02, \mathrm{SD}=6.93)$ in comparison to the *below median* *awareness of error* score group $\left(M=-3.02, \mathrm{SD}=3.06\right), t\left(10\right)=-0.97, p=0.355$ for N135 peak (see Fig. 5b; Table 3). On the other hand, for N400 peak, the *above median awareness of error* score group showed a weaker training-ERN response $(M=1.72, \mathrm{SD}=5.60)$ in comparison to the* below median awareness of error* score group $\left(M=-4.34, \mathrm{SD}=5.75\right), t\left(10\right)=1.85, p=$0.094 (see Fig. 5b; Table 3). A similar mixed pattern is also evident from the central regions of the topographical maps shown in Fig. 5b. However, the difference in the ERN responses at the two latencies did not show significance (see Table 3).

**Table 3:** Mean, standard deviation and independent-samples *t* test statistics of difference in training-ERN peaks of patients with *interest/enjoyment* state score *above median* and *below median*, and *awareness of error* state score *above median* and *below median*

State	Peak amplitude latency (ms)	*Above median*/*below median* group	Mean ± SD	*T*-test statistics (-inter)
*Interest/enjoyment*	100	Above	2.74 ± 3.95	*t*(10) = 2.68, *p* = 0.023
		Below	–1.65 ± 0.78	
	410	Above	–5.43 ± 4.55	*t*(10) = –2.69, *p* = 0.023
		Below	2.28 ± 5.35	
*Awareness of error*	135	Above	–6.02 ± 6.93	*t*(10) = –0.97, *p* = 0.355
		Below	–3.02 ± 3.06	
	410	Above	1.72 ± 5.60	*t*(10) = 1.85, *p* = 0.094
		Below	–4.34 ± 5.75	

## Discussion

The goal of this study was to assess whether the ERN signal associated with the novel training-ErrP signal is affected by the psychological and cognitive states of stroke patients, and to evaluate the possibility of estimating participants' psychological and cognitive states using the amplitude of their training-ERN signal. The psychological and cognitive states included in this study were motivation to participate in the experiment, perceived effort, perceived pressure, and awareness of uncompleted exercise trials. The present study found strong evidence for an association between the amplitudes of training-ERN peaks and participants’ scores on the MEPA states. Strong correlations among the peaks of the training-ERN signal and the MEPA states indicate that the states can be estimated using the amplitude of the patient’s training-ERN peaks.

The *interest/enjoyment* scale represents the intrinsic motivation of the participants to participate in the experiment [25], and the *pressure/tension* scale represents the stress of the participants to participate in the experiment [25]. The *interest/enjoyment* and *pressure/tension* correlation results showed higher brain activations by means of stronger training-ERN response for higher motivation levels and weaker training-ERN responses for higher stress levels which is a negative predictor for motivation levels. Moore et al. [19] have linked motivation with mental fatigue which affects the task performance of participants. As long as one feels that the perceived rewards of executing a task are sufficient, the motivation to participate in the task remains [19]. By contrast, insufficient rewards diminish the motivation of the participants, which results in disengagement from the task [19]. Higher motivation and thereby higher task engagement results in an enhanced action monitoring system of the brain, which results in stronger ERN signals, as observed in the case of association between training-ERN and *interest/enjoyment* scores in the present study. These results are in keeping with previous observational studies which set out the association between motivation levels and typical-ERN signal amplitudes [15, 16, 32]. In addition, previous studies have also observed an impaired action monitoring system in participants in whom symptoms of stress and depression exist [12, 13, 18]. The results of these studies are in agreement with the association between the perceived *pressure/tension* scores and training-ERN amplitudes observed in the present study.

The perceived effort of the participants to execute the task represents a conscious sensation of how hard the participant had to drive their impaired upper-limb to complete the rehabilitation exercise in the given time. Greater effort to carry out a task shows higher cognitive resource engagement and lower mental fatigue [15]. A number of previous studies have linked lower effort and higher mental fatigue with weak typical-ERN signals, and *vice versa* [19, 33]. Boksem et al. [15] conducted an experiment in which participants performed a given task continuously for 2 h. They observed a change in the task performance with ongoing fatigue, which was accompanied by a substantial decrease in ERN amplitude, indicating that action monitoring of the brain is impaired in fatigued participants. In the present study, a higher perceived *effort/importance* score was observed to be associated with a significant increase in P100 amplitude and a substantial increase in the N400 amplitude of the training-ERN signal, which is consistent with the literature. A higher effort has also been linked with a higher perception of rewards, which results in enhanced action monitoring, which increases the ERN amplitude [21]. Nevertheless, a higher perception of reward comes down to individual perception towards the benefit of rehabilitation exercises or the stroke rehabilitation program in general; which can be investigated in the future.

A positive deflection, i.e., the Pe peak of a typical-ERN signal, has been linked with cognizance of the error made [34–36]. In other words, when the participants are unaware of the error, Pe has been observed to be strongly diminished. However, a negative deflection, i.e., the Ne peak of a typical-ERN signal, is unaffected by this unawareness [34–36]. The present study has revealed an increase in the amplitude of the N135 peak with an increase in the score of *awareness of error* state, though not statistically significant; on the other hand, the P100 peak has remained largely unaffected. Interestingly, this behavior of training-ERN signal is opposite to that of a typical-ERN signal. In training-ERN signal, an unaffected P100 and stronger N135 peaks occur; whereas in a typical-ERN signal, unaffected Ne and stronger Pe peaks occur, in response to greater awareness of error [34–36]. Notably, Kumar et al. [7] observed a reverse polarity in the ERN signal associated with the training-ErrP signal in comparison to the polarity observed in typical-ERN signals in the literature [11, 34–36], which supports the opposite behavior observed in the present study. This result suggests the attribution of P100 deflection of the training-ERN signal with Ne deflection seen in typical-ERN signals, as well as the attribution of N135 deflection of the training-ERN signal with the Pe deflection seen in typical-ERN signals [7, 34–36]. The decrease in the amplitude of the N400 peak with an increase in *awareness of error* score can be attributed to the methodological differences in relation to the training-ERN signals. The observed relationship between the training-ERN signal and the typical-ERN signal can assist in understanding the neural mechanism behind the training-ERN signal and in comparing ERN signals of various task modalities [11].

Although a handful of correlations among the peaks of the training-ERN signal and the psychological and cognitive states showed statistical significance, the majority of correlations follow a consistent trend supported by the literature. These correlations indicate the possibility of developing a training-ERN based algorithm that can estimate patients’ psychological and cognitive states through the amplitude of their training-ERN signals. With the help of such algorithms, a dynamic rehabilitation program can be established in which the characteristics of the program, such as exercise type, difficulty and duration, can be altered to keep the states such as motivation, effort and engagement high, which can improve the rate and amount of recovery from stroke disabilities [8, 22, 24]. Previous studies have also explored the use of the automatic nervous system (ANS) to develop a dynamic rehabilitation program. For instance, Koenig et al*.* [37] and Novak et al*.* [38] used inputs from ANS such as skin-conductance level, heart rate and skin temperature to estimate the engagement and arousal level of stroke patients while performing rehabilitation movements, and proposed their use in developing a closed-loop auto-adaptive rehabilitation program. Such systems have been shown to promote the engagement level and participation, and improve the overall user experience [9, 10, 39], which can increase the rate and amount of recovery from stroke disabilities [8, 22, 24]. However, stroke patients often show long-lasting abnormalities in ANS, which can alter the functioning of ANS-based auto-adaptive rehabilitation programs [40]. In addition, for every ANS function included in the auto-adaptive rehabilitation program, such as skin-conductance level and heart rate, the complexity of the rehabilitation system increases. On the other hand, the psychophysiological measurements using the training-ERN signal does not increase the complexity of the system, given that EEG measurement and processing already form a part of the training-ErrP-led AAN robotic stroke rehabilitation system [7]. However, the stroke may influence the training-ERN signal depending upon the location of the brain lesion, which is an important issue for future research. Nevertheless, the present study provides a stepping stone for the use of training-ERN signals to estimate the psychological and cognitive states of the stroke patients; while substantial work is needed before such a system can be used in the real world. For instance, the feasibility to predict the state-of-mind of stroke patients in single trials of training-ERN signals needs to be determined using classification or regression models, as the group-level analyses alone, as conducted in this study, do not guarantee single-trial prediction feasibility. However, the determination of the true state-of-mind of stroke patients for every single trial is quite challenging, especially with questionnaires, at which more work is needed before patients’ psychological and cognitive states can be integrated in robot-control algorithms. Furthermore, factors such as the *error trials* rate and recovery level of stroke patients can also influence the training-ERN signal as well as the state-of-mind of stroke patients, and need to be evaluated through longitudinal studies with a higher number of participants, before such a training-ErrP-led dynamically adaptive AAN robotic stroke rehabilitation system can be used in practice in real-world applications.

## Conclusion

Training-ERN is a new type of ERN signal recently observed by our research group, which adds a novel modality to develop AAN robotic stroke rehabilitation programs. The present research aimed to determine the association between the training-ERN signal and the psychological and cognitive states of the participants, which is a first such attempt to the best of the author’s knowledge. In this study, experiments were conducted on stroke patients which involved performing a physical rehabilitation exercise and a questionnaire to measure participants’ subjective experience in respect of four states while performing the rehabilitation exercise: motivation to participate in the experiment; perceived effort; perceived pressure; and awareness of uncompleted exercise trials. The study has identified strong correlations among the amplitude of the training-ERN signal and the psychological and cognitive states of the participants; which indicates the possibility of estimating the said states using the amplitude of their training-ERN signal. The new understandings gained from this study would help in developing a training-ERN-based, dynamically adaptive AAN robotic stroke rehabilitation system which can alter its characteristics in order to keep the participants’ psychological and cognitive states such as motivation, effort and engagement high, which can drive the neuroplasticity of the brain and improve the rate of recovery of stroke patients from their motor impairment. In future, the single-trial prediction ability of the training-ERN signals to predict the state-of-mind of stroke patients will be evaluated.

## Data Availability

The datasets generated and analyzed during the current study are available from the corresponding author on request.
